# Identification of Immune-Related Risk Signatures for the Prognostic Prediction in Oral Squamous Cell Carcinoma

**DOI:** 10.1155/2021/6203759

**Published:** 2021-08-25

**Authors:** Chen Zou, Dahong Huang, Haigang Wei, Siyuan Wu, Jing Song, Zhe Tang, Xia Li, Yilong Ai

**Affiliations:** Foshan Stomatological Hospital, School of Medicine, Foshan University, Foshan, Guangdong, China

## Abstract

**Background:**

Oral squamous cell carcinoma (OSCC) is the most common type of oral cancer, which remains a major cause of morbidity and mortality in patients with head and neck cancers. However, the critical immune-related signatures and their prognostic values have rarely been investigated.

**Materials and Methods:**

Gene differential analysis was used to measure the differences of gene expression between the groups. Correlation analysis was used to assess the association between the gene expression levels and immune-related risk score/DNA methylation levels. The gene set enrichment analysis (GSEA) was used to identify the pathways or cell types enriched by those identified differentially expressed genes (DEGs).

**Results:**

In this study, we identified four immune-related gene signatures, including *CTSG*, *TNFRSF4*, *LCORL*, and *PLAU*, that were significantly associated with the overall survival in OSCC patients from the Cancer Genome Atlas (TCGA) OSCC cohort. Moreover, these four immune-related signatures were differentially expressed between the OSCC and nontumor tissues. The two groups (high and low risk) stratified by the immune-related risk scores had significantly different OS and mortality rates. The gene expression patterns and prognostic values of these immune-related signatures were also verified in two independent validation cohorts. Furthermore, the downregulated genes in the high-risk group (which were also upregulated in the low-risk group) were significantly enriched in the cell type-specific signatures of type 2 T helper cell (Th2), plasmacytoid dendritic cell (pDC), and memory B cell. In contrast, the upregulated genes in the high-score group were enriched in growth factor receptor-related signaling pathways, such as the VEGFA-VEGFR2 signaling pathway, PI3K-Akt signaling pathway, focal adhesion-PI3K-Akt-mTOR signaling pathway, and PDGF pathway, suggesting that those pathways were inversely correlated with immune cell infiltration.

**Conclusion:**

In summary, the immune-related signatures had the potential for predicting the risk of OSCC patients. Moreover, the present study also improved our understanding of the association between the growth factor receptor pathways and immune cell infiltration in OSCC.

## 1. Introduction

Oral squamous cell carcinoma (OSCC) constitutes a huge proportion of head and neck malignancies, posing a growing threat to the global public health [1]. According to the GLOBOCAN 2020 estimates, there are approximately 377713 new cases of lip and oral cavity cancer and 177757 related deaths in 2020 and its high incidence in specific areas is considered as a reflection of certain etiological factors, such as betel nut chewing, smoking, alcohol intake, excessive sunlight exposure, and human papillomavirus (HPV) infection [1, 2]. Of note, it is believed that head and neck squamous cell carcinoma (HNSCC) can be divided into HPV-positive and HPV-negative subgroups based on the presence or absence of HPV infection and OSCC is no exception [3]. Improved prognoses are observed in HPV-positive head and neck cancer patients with a controlled load of tumor-type HPV DNA during therapy, and differences are appreciated in the immune profiles of HPV-positive and HPV-negative patients, as increased expression of a T-regulatory cell (T-regs) marker gene and decreased expression of a M2 protumorigenic macrophage marker gene were observed in HPV-positive HNSCC compared to HPV-negative tumors [4], hinting that further exploration of the tumor microenvironment in OSCC shall benefit the identification of prognostic markers and therapeutic targets [5, 6].

The tumor microenvironment (TME) comprises multiple cell types such as immune cells, endothelial cells, pericytes, and fibroblasts embedded in the extracellular matrix [7], and TME is often considered a determinative player in the development and progression of cancer, influencing T cell response against tumor-associated antigens and response to immunotherapy [8, 9]. Utilizing gene expression data, a recent study has reported 6 immune subtypes in squamous cell carcinomas, each with identical patterns of immune-related gene expression, and the survival outcomes significantly varied across different subtypes, where similar to previously published research findings, higher levels of immune infiltration are observed to be associated with favorable prognosis, possibly via upregulated pathways that are involved in chemokine and cytokine signaling, T cell survival following inflammation, antigen presentation, and tumor progression [10, 11]. Those pathways include IL-2, IL-6, IL-10, CD40, MAPK/ERK, TGF*β*, JAK/STAT, and AKT/mTOR pathways; all of which are frequently addressed in varied cancer types. Altered composition of immune cell populations can be indicative of patients' prognoses in HNSCC, as reported in another study. It is found that the fractions of resting memory CD4 T cells, M1 macrophages, resting dendritic cells, resting mast cells, monocytes, and eosinophils in HNSCC tissues are associated with HNSCC progression, while lower infiltration of memory CD4 T cells would lead to worse prognosis [12]. However, key immune-related signatures and their prognostic values in OSCC still need further exploration, which can deepen our understanding toward OSCC and help identify certain patients that could potentially benefit from immunotherapy.

## 2. Materials and Methods

### 2.1. Data Acquisition

The gene expression data from the Cancer Genome Atlas (TCGA) [13] and validation cohort were downloaded from TCGA data portal (https://portal.gdc.cancer.gov/), ArrayExpress (https://www.ebi.ac.uk/arrayexpress/, accession: E-MTAB-8588 [14]), and Gene Expression Omnibus (GEO, https://www.ncbi.nlm.nih.gov/gds, GSE31056). The OSCC samples with primary sites at the base of tongue, oropharynx part, oral cavity, hypopharynx, gum, palate, and lip were collected for the data analysis in this study. The clinical characteristics of the patients from the two OSCC cohorts were summarized in Table 1. The RNA-seq- and microarray-based gene expression profiles were prenormalized to log2 FPKM or background corrected and quantile normalized. The DNA methylation data was also obtained from the TCGA data portal and normalized as the beta value. The signature genes of 28 immune cell types were collected from previous studies [15, 16] and merged for our data analysis.

### 2.2. Feature Selection, Cox Regression Model Construction, and Risk Stratification

A total of 782 immune-related genes were collected from the previous studies [15, 16]. To identify a clinically translatable immune gene signature, we performed Lasso-penalized Cox regression analysis on these genes. We used Cox proportional hazard regression analysis to evaluate the capabilities of immune-related signatures in predicting overall survival (OS) in OSCC patients. The OSCC patients were stratified into high- and low-risk groups using the median value of the immune-related risk scores as a threshold. Kaplan-Meier (KM) analysis was performed to determine survival outcomes. The KM curves were plotted with the R survminer package (https://cran.r-project.org/web/packages/survminer/index.html).

### 2.3. The Differential Gene Expression Analysis

The differential gene expression analysis was conducted using the R limma package [17]. Those genes with a false discovery rate (FDR) < 0.05 and a mean difference higher than twofold were considered as differentially expressed genes (DEGs).

### 2.4. The Pathway Enrichment Analysis

The pathway enrichment analysis was performed using the hypergeometric test. The genes associated with certain pathways from KEGG [18], Reactome [19], and WikiPathway [20] were downloaded using the R msigdbr package [21]. This analysis was implemented in the R clusterProfiler package [22]. The adjusted *P* value < 0.05 was used as the threshold to identify pathways enriched by the differentially expressed genes.

### 2.5. Tissue Specimens

Three pairs of fresh OSCC and adjacent normal tissues were collected from patients in the Foshan Stomatological Hospital, which was approved by the Human Research Ethics Committee of this hospital. The written informed consent was collected from each patient. All samples were stored at −80°C for the following experiments.

### 2.6. Quantitative Real-Time Polymerase Chain Reaction (qPCR)

The tissue samples were first lysed using a TRIzol reagent (Invitrogen, USA). The reverse transcription was performed using a RevertAid First Strand cDNA Synthesis Kit (Thermo Fermentas, USA). The mRNA expression was quantitatively analyzed using an ABI StepOnePlus (StepOnePlus™), with GAPDH as an internal reference. The primers for CTSG, TNFRSF4, LCORL, PLAU, PDGFA, PDGFB, and GAPDH were listed in Supplementary Table S1. The normalized RNA expression levels of the samples were calculated as the expression value of the target RNA divided by that of the internal reference.

### 2.7. Statistical Analysis

The comparison between two groups was tested by a two-sample Wilcoxon test. The pairwise Wilcoxon test was used to test the difference between multiple groups. The *P* values < 0.05, 0.01, and 0.001 were represented by ^∗^, ^∗∗^, and ^∗∗∗^, respectively.

## 3. Results

### 3.1. Identification of Immune-Related Prognostic Signatures in OSCC

**Figure 1:**
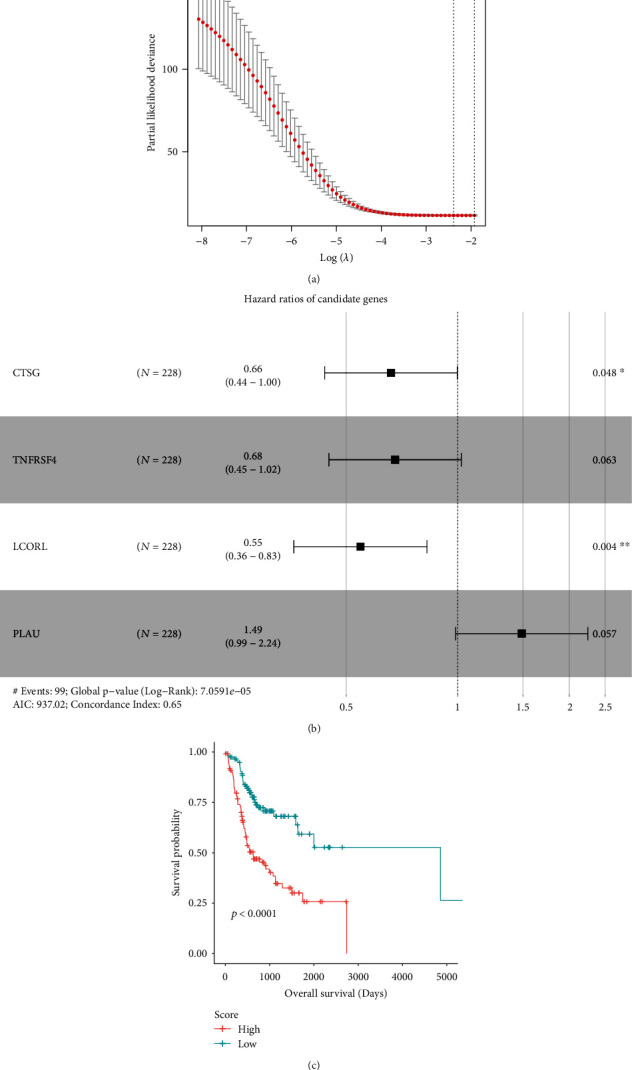
Feature selection and model construction for overall survival prediction in OSCC. (a) The estimation of the number of genes used for multivariate Cox analysis. The *y*-axis represents the partial likelihood deviance. The vertical line on the left represents the four genes used for model construction. (b) The forest plot represents the hazard ratio and 95% confidence interval for the four immune-related signature genes. (c) The Kaplan-Meier (KM) curves for the high- and low-score groups of OSCC patients in the TCGA cohort.

To shed light on the immune landscape of OSCC, we first collected 228 OSCC samples from head and neck squamous cell carcinoma (HNSCC) of the TCGA cohort, including 53 that occurred in the floor of mouth, 20 in the base of tongue, 11 in the gums, 10 in the oropharynx, 5 in the palate, 3 in the lips, and 126 in other and unspecified parts of the tongue, as well as 35 normal samples adjacent to tumors (NATs). The signature genes of 28 immune cell types were collected from previous studies [15, 16]. In all, the immune signatures contained 782 genes representing 28 microenvironment cell types. Subsequently, Lasso-penalized Cox regression analysis was performed for variable selection in the Cox model. Using the lambda.min cutoff threshold, we identified four immune-related gene signatures, including *CTSG*, *TNFRSF4*, *LCORL*, and *PLAU*, that were significantly associated with overall survival in OSCC patients (Figure 1(a)). The multivariate Cox regression analysis on the four genes indicated that *CTSG*, *TNFRSF4*, and *LCORL* were positively correlated with OS, while *PLAU* was negatively correlated with OS in OSCC (Figure 1(b)). It is worth noting that the *TNFRSF4* and *PLAU* exhibited no significant association with OS for a given *P* value of 0.05, but their *P* values were very close to this threshold. The multivariate model achieved a high global statistical significance (log-rank test, *P* value = 7.06*e* − 5). Furthermore, we derived a score based on the four-gene signature, which was termed as an immune-related risk score and stratified the OSCC patients into high- and low-risk groups. As shown in Figure 1(c), the high-risk group had significantly shorter OS than the low-risk group (median of OS: 625 vs 4856 days, log-rank test, *P* value < 0.0001). Notably, there were significant differences of mortality between the high-risk and low-risk groups (Figure 1(c), proportion of deceased patients: 58.6% (*n* = 111) vs 29.1% (*n* = 117), Pearson's chi-squared test, *P* value < 0.0001). These analyses indicated that the immune-related signatures had the potential to predict the prognosis of OSCC patients.

### 3.2. The Clinical Association of the Immune-Related Prognostic Signatures in OSCC

**Figure 2:**
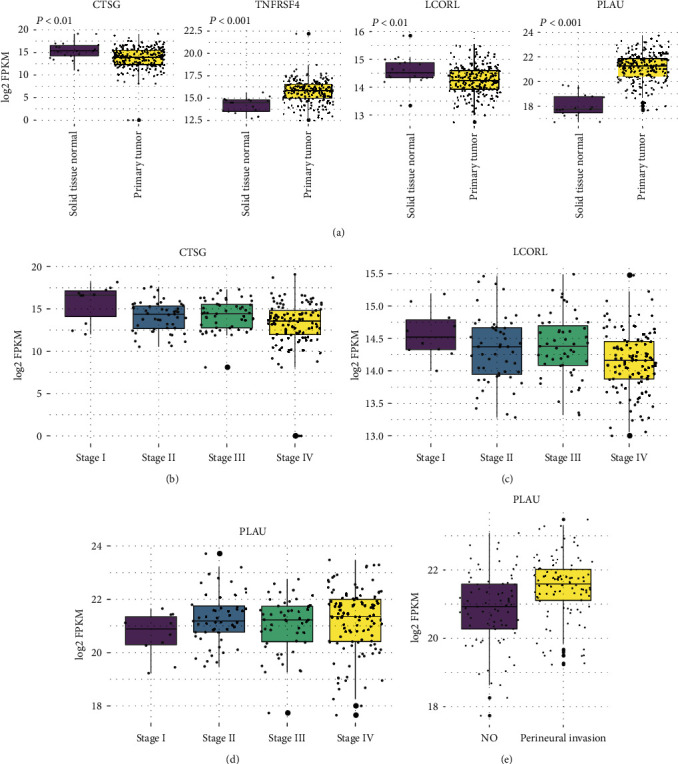
Differential gene expression of the four immune-related gene signatures between tumor and adjacent normal tissues, TNM stages, or tumors with and without perineural invasion. (a) The gene expression patterns of CTSG, TNFRSF4, LCORL, and PLAU between the OSCC and adjacent normal tissues in the TCGA cohort. The differential expression of (b) CTSG, (c) LCORL, and (d) PLAU between the OSCC patients with distinct TNM stages. (e) PLAU is expressed higher in OSCC with perineural invasion than those without.

As the four immune-related genes were identified as prognostic signatures in OSCC, we then tested the association between their expression levels and other clinical characteristics. The comparison of gene expressions in tumor and normal tissues revealed that *CTSG* and *LCORL* were downregulated, while *TNFRSF4* and *PLAU* were upregulated in OSCC tissues (Figure 2(a)). Consistently, the expression levels of two downregulated genes in OSCC, *CTSG* and *LCORL*, were decreased along with the progressed TNM staging, especially in patients with metastatic diseases (Figures 2(b) and 2(c), analysis of variance (ANOVA), *P* value < 0.05). In contrast, a higher expression of the upregulated gene, PLAU, was observed in patients of advanced stages (Figure 2(d), analysis of variance (ANOVA), *P* value < 0.05). In addition, PLAU was also expressed higher in OSCC patients with perineural invasion as compared with those without (Figure 2(e)), suggesting that a high expression of PLAU might indicate a poor prognosis in OSCC.

### 3.3. Independent Validation of the Immune-Related Prognostic Signatures

**Figure 3:**
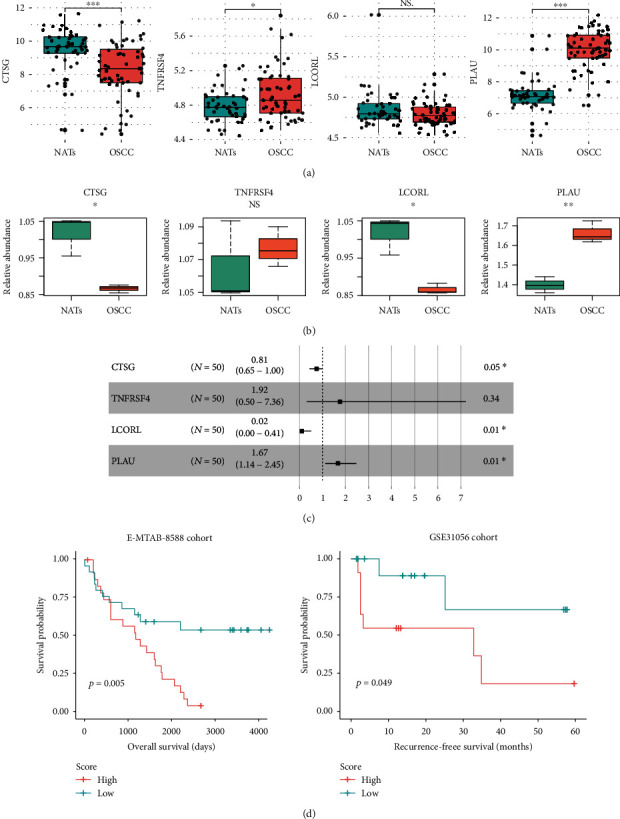
The validation of the expression patterns and prognostic values of the immune-related gene signatures. (a) The gene expression patterns of CTSG, TNFRSF4, LCORL, and PLAU between the OSCC and adjacent normal tissues in the E-MTAB-8588 cohort. (b) The differential expression levels of CTSG, TNFRSF4, LCORL, and PLAU in three pairs of OSCC and NATs by the qPCR method. (c) The forest plot displays the hazard ratios and confidence intervals of the immune-related signatures by univariate Cox regression analysis in the E-MTAB-8588 cohort. (d) Kaplan-Meier (KM) curves for the high- and low-score groups of OSCC patients in E-MTAB-8588 and GSE31056 cohorts.

To further validate the expression pattern and prognostic value of the four immune-related prognostic signatures, we collected two independent datasets from the ArrayExpress database (accession ID: E-MTAB-8588) with 50 OSCC and 57 NAT samples and Gene Expression Omnibus (GEO) (GSE31056) with 23 OSCC samples. The differential expression analysis of the four immune-related genes revealed that CTSG was significantly downregulated in OSCC, while TNFRSF4 and PLAU were upregulated as compared with the NATs in the E-MTAB-8588 cohort (Figure 3(a)). Even though the downregulation of LCORL was not significant (Figure 3(a), *P* value > 0.05), its expression was still decreased in OSCC to a certain extent. To verify such differential expression, we collected 3 pairs of OSCC and NAT tissues and conducted quantitative real-time polymerase chain reaction (qPCR) to quantify the four immune-related genes. Consistently, CTSG, LCORL, and PLAU were validated to be differentially expressed (Figure 3(b)). Moreover, we also conducted univariate Cox regression analysis on the four genes in the validation cohort. Consistently, the expressions of CTSG and LCORL were also observed to be positively correlated with OS, while PLAU expression was negatively correlated with OS in OSCC (Figure 3(c)). Exceptionally, the expression of TNFRSF4 was not significantly associated with OS in the validation cohort (Figure 3(c)). Furthermore, we also calculated the immune-related risk score for the OSCC patients in the validation cohorts using the expression levels of the four genes. Likewise, the OSCC samples in the two validation cohorts were also stratified into high- and low-risk groups. The comparison of the OS between the two groups further confirmed that the high-risk group had significantly shorter OS and higher mortality than the low-risk group (Figure 3(d), proportion of deceased patients: 88% (*n* = 25) vs 44% (*n* = 25), log-rank test, *P* value = 0.005) in the E-MTAB-8588 cohort. Even though the sample size of the GSE31056 cohort was small (*n* = 23), the high-risk group still had significantly shorter recurrence-free survival (RFS) than the low-risk group (Figure 3(d), *P* value < 0.05). These results indicated that the four immune-related signatures showed a similar expression pattern and prognostic value between the TCGA and validation cohorts.

### 3.4. Differential Expression of Immune-Related Signatures between Immune-Related Risk Groups

**Figure 4:**
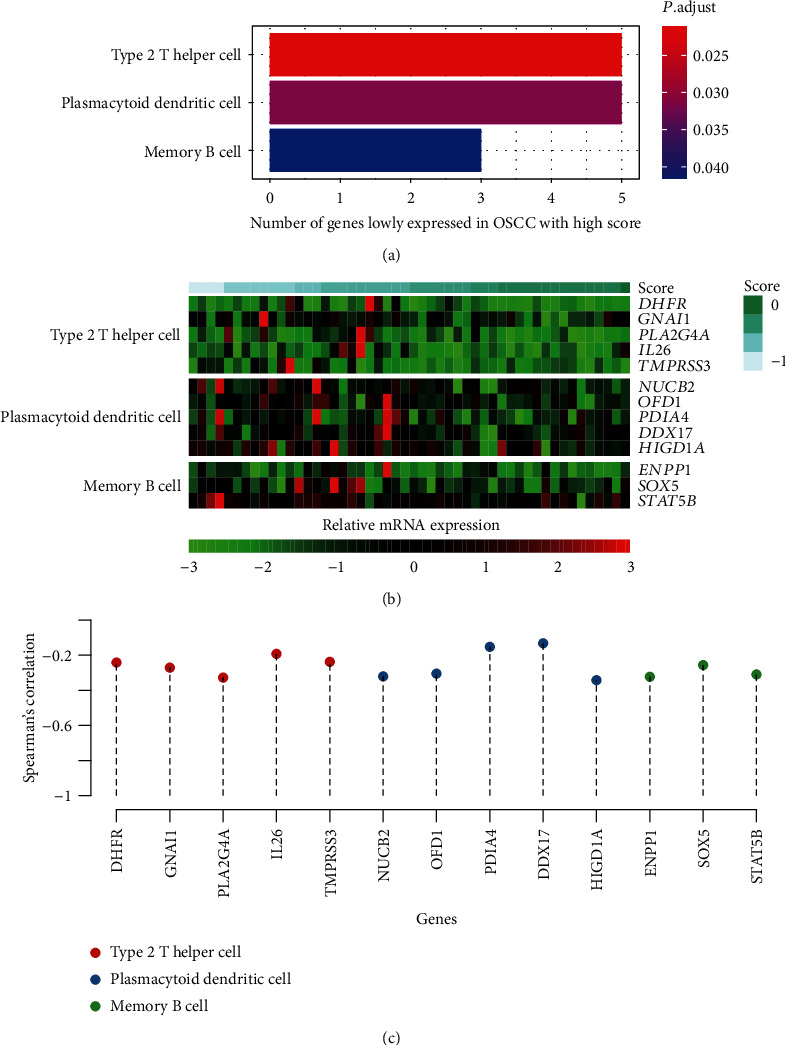
The immune cell-specific signatures and immune cells associated with immune-related risk stratification. (a) The pathways enriched by the downregulated genes in the high-score group and corresponding gene numbers. The colors represent the statistical significance (adjusted *P* value). (b) The gene expression profiles of the immune cell-specific signatures. The samples were ordered by the immune-related risk scores. (c) The Spearman's correlation between the immune cell-specific signatures and immune-related risk scores. The colors represent the pathways that the genes were involved in.

To further illuminate the relationship between the four immune-related signatures and immune cells infiltrating into OSCC tissues, we conducted differential gene expression analysis between the high- and low-risk groups. The downregulated genes in the high-risk group were significantly enriched in the cell type-specific signatures of type 2 T helper cell (Th2), plasmacytoid dendritic cell (pDC), and memory B cell (Figure 4(a), Fisher's exact test, adjusted *P* value < 0.05). In light of the better prognoses in the low-risk group, we speculated that a higher abundance of these immune cell types might have inhibitory effect on tumor growth in OSCC. Consistently, these cell type-specific genes were expressed higher in samples of low risk scores (Figure 4(b)) and the correlation analysis between immune-related risk scores and the expression levels of those genes confirmed that they had negative correlations (Figure 4(c)). These results suggested that the high-risk group, characterized by worse prognoses, had lower infiltrating levels of type 2 T helper cell, plasmacytoid dendritic cell, and memory B cell, along with decreased expression levels of cell type-specific signatures.

### 3.5. The Regulation of Immune-Related Prognostic Signatures and Pathways

**Figure 5:**
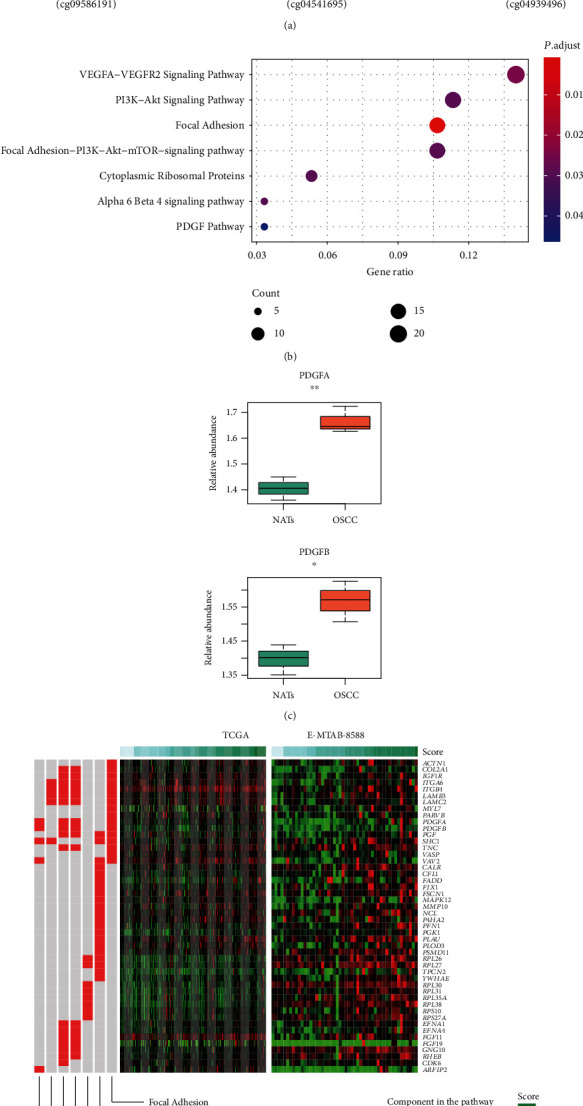
The epigenetic regulation of the four immune-related genes and pathways associated with immune-related risk stratification. (a) The correlation between the promoter DNA methylation and gene expression levels of TNFRSF4, LCORL, and PLAU. (b) The pathways enriched by the upregulated genes in the high-score group. (c) The expression profiles of genes involved in the pathways. The OSCC samples were ordered by immune-related risk scores. (d) The differential expression levels of PDGFA and PDGFB in the three pairs of OSCC and NATs by the qPCR method.

To further investigate the downstream regulators of the four immune-related prognostic signatures, we conducted correlation analysis between the RNA expression levels and DNA methylation levels in the TCGA cohort. The pairwise correlation analysis revealed that TNFRSF4, LCORL, and PLAU were negatively correlated with DNA methylation levels of their corresponding promoter CpG sites (Figure 5(a), *P* value < 0.05). Moreover, we also verified the DNA methylation and RNA expression using two public datasets of OSCC (GSE41117 and GSE75539). As shown in Figure S1, the methylation levels of the three genes were negatively correlated with RNA expression levels. Notably, such negative correlations for TNFRSF4 and PLAU were statistically significant in GSE41117 and GSE75539 (*P* value < 0.05), respectively. Furthermore, we also identified 279 genes jointly upregulated in the high-risk groups of both TCGA and E-MTAB-8588 cohorts. The pathway enrichment analysis helped us identify growth factor-receptor-related signaling pathways, such as the VEGFA-VEGFR2 signaling pathway, PI3K-Akt signaling pathway, focal adhesion-PI3K-Akt-mTOR signaling pathway, and PDGF pathway; all of which were significantly enriched by those upregulated genes (Figure 5(b), adjusted *P* value < 0.05). Consistently, the components involved in these pathways were positively correlated with the immune-related risk scores (Figure 5(c), correlation test, *P* value < 0.05). Furthermore, the qPCR analysis of the two platelet-derived growth factors (PDGFs), PDGFA and PDGFB, revealed that the three OSCC tissues had higher expression levels than the paired NATs (Figure 5(d), *t*-test, *P* value < 0.05). These results indicated that immune-related signatures were correlated with growth factor-receptor-related signaling pathways.

## 4. Discussion

Oral squamous cell carcinoma (OSCC) is the most common type of oral cancer, which remains a major cause of morbidity and mortality in patients with head and neck cancers. However, the critical immune-related signatures and their prognostic values in OSCC have rarely been discussed. In this study, we identified four immune-related gene signatures, including *CTSG*, *TNFRSF4*, *LCORL*, and *PLAU*, that were significantly associated with overall survival in OSCC patients. The multivariate Cox regression analysis on the four genes indicated that *CTSG*, *TNFRSF4*, and *LCORL* were positively correlated with OS, while *PLAU* was negatively correlated with OS in OSCC. It should be noted that the four immune-related signatures were differentially expressed between the OSCC and NATs. *CTSG* was reported as a potential immune-related biomarker in OSCC by a recent study [23], and it has also been found to be associated with the survival of other cancer types such as soft tissue sarcoma [24], muscle-invasive bladder cancer [25], and node-negative breast cancer [26]. Consistently, *TNFRSF4* and *PLAU* exhibited a potential association with OS in HNSCC [27, 28]. Based on our collected data, *LCORL* was characterized as a marker gene of *γδ* T cell and encoded a transcription factor, according to a previous study [29]. The OSCC patients were stratified into two groups (high vs low risk). The high-risk group had significantly shorter OS and higher mortality than the low-risk group. The gene expression patterns and prognostic values of these immune-related signatures were also verified in two independent validation cohorts.

To have a deeper understanding of the biological differences between these two groups, we compared the gene expression profiles between the OSCC patients of the two groups. The downregulated genes in the high-risk group (which were also upregulated in the low-risk group) were significantly enriched in the cell type-specific signatures of type 2 T helper cell (Th2), plasmacytoid dendritic cell (pDC), and memory B cell. It is well recognized that Th2, pDC, and memory B cell have inhibitory effects on cancer growth and progression [30–32]. As DNA methylation plays a key role in anticancer immune response (24212778), we tested the correlation between the DNA methylation and gene expression levels of the four immune-related signatures and found that *TNFRSF4*, *LCORL*, and *PLAU* were negatively correlated with DNA methylation levels of their corresponding promoter CpG sites, suggesting that those genes were regulated by DNA hypermethylation. In contrast, the upregulated genes in the high-risk group were enriched in growth factor-receptor-related signaling pathways, such as the VEGFA-VEGFR2 signaling pathway, PI3K-Akt signaling pathway, focal adhesion-PI3K-Akt-mTOR signaling pathway, and PDGF pathway, suggesting that those pathways were inversely correlated with immune cell infiltration. Furthermore, we also verified the upregulations of PDGFA and PDGFB in three OSCC samples using the qPCR method. In accordance with this finding, VEGFA-VEGFR2, PI3K-Akt, and mTOR signaling could regulate the immune cell infiltration and inhibit immune function [33–35].

In summary, we have identified *CTSG*, *TNFRSF4*, *LCORL*, and *PLAU* as key immune-related signatures in predicting the OS of OSCC. The comparison of the gene expression profiles between the two immune-related groups revealed that critical immune cell types, including Th2, pDC, and memory B cells, might have anticancer properties in OSCC, while the growth factor-receptor-related signaling pathways like VEGFA-VEGFR2, PI3K-Akt, mTOR, and PDGF signaling pathways might inhibit the immune cell infiltration and promote tumor progression. Collectively, the proposed immune-related signatures had the potential to be applied in predicting the risk of OSCC patients. Moreover, the present study also improved our understanding of the association between the growth factor-receptor pathways and immune cell infiltration in OSCC.

## Figures and Tables

**Table 1:** The clinical characteristics of OSCC patients from TCGA, E-MTAB-8588 cohorts, and GSE31056.

Factors	TCGA (*n* = 228)	E-MTAB-8588 (*n* = 59)	GSE31056 (*n* = 23)	*P* value
Gender				0.0073
Male	164 (71.93%)	53 (89.83%)	NA	
Female	64 (28.07%)	6 (10.17%)	NA	
Stage				0.11
I	10 (4.39%)	6 (10.17%)	0 (0%)	
II	50 (21.93%)	7 (11.86%)	4 (30.77%)	
III	51 (22.37%)	12 (20.34%)	1 (7.69%)	
IV	110 (48.25%)	33 (55.93%)	8 (53.84%)	
HPV status				0.83
Positive	9 (20.45%)	12 (20.34%)	NA	
Negative	44 (79.55%)	47 (79.66%)	NA	
Age	59.36 + 12.72	58.40 + 11.40	NA	0.68
Smoking history				0.036
Yes	164 (72.89%)	52 (88.14%)	NA	
No	61 (27.11%)	7 (11.86%)	NA	
Primary sites				0.88
Base of the tongue	20 (8.77%)	20 (33.90%)	NA	
Gum	11 (4.82%)	0 (0%)	NA	
Oropharynx	10 (4.39%)	15 (25.42%)	NA	
Palate	5 (2.19%)	0 (0%)	NA	
Lip	3 (1.32%)	0 (0%)	NA	
Other and unspecified parts of the tongue	126 (55.26%)	24 (40.68%)		
Vital status				0.12
Dead	99 (43.42%)	33 (55.93%)	NA	
Alive	129 (56.58%)	26 (44.07%)	NA	

## Data Availability

The gene expression and clinical data used to support this study are available at the Cancer Genome Atlas (TCGA, https://portal.gdc.cancer.gov/), ArrayExpress, and Gene Expression Omnibus (GEO) databases. These prior studies (and datasets) are cited at relevant places within the text as references.

## Supplementary Materials

Supplementary 1 Table S1. The primers of the genes for qPCR.

Supplementary 2 Figure S1. The correlation between the promoter DNA methylation and gene expression levels of TNFRSF4, LCORL, and PLAU in the validation datasets (A: GSE41117 and B: GSE75539).
