# Supplementary material for: Identification of Immune-Related Risk Signatures for the Prognostic Prediction in Oral Squamous Cell Carcinoma
Source: J Immunol Res. 2021 Aug 25;2021:6203759. doi: 10.1155/2021/6203759 (PMC8420972; doi:10.1155/2021/6203759)
Supplement: Supplementary 1 — Table S1. The primers of the genes for qPCR. [file 6203759.f1.docx]

| **Table S1. The primers of the genes for qPCR** | | |  |
| --- | --- | --- | --- |
| Gene | Forward primer | Reverse primer | PubMed ID |
| CTSG | TCCTGGTGCGAGAAGACTTTG | GGTGTTTTCCCGTCTCTGGA | 16652140 |
| TNFRSF4 | ACAACGACGTGGTCAGCTCCAA | CAGCGGCAGACTGTGTCCTGT | 30944836 |
| LCORL | CTTTATGGACCACGGCTACGA | TTTTCATCCATAGACCAGTCAGTCA | 23418579 |
| PLAU | CACACACTGCTTCATTGATTACCCA | TTTTCCACCTCAAACTTCATCTCCC | 33574243 |
| PDGFA | GAACTCACGGTGGCTGCTG | GAACTCACGGTGGCTGCTG | 16470538 |
| PDGFB | TGAACGTGGTCAACCTGT | ACGAGGTCCATGTAGCT | 16470538 |
| GAPDH | GGAGCGAGATCCCTCCAAAAT | GGCTGTTGTCATACTTCTCATGG |  |
