# Supplementary material for: Identification of Immune-Related Risk Signatures for the Prognostic Prediction in Oral Squamous Cell Carcinoma
Source: J Immunol Res. 2021 Aug 25;2021:6203759. doi: 10.1155/2021/6203759 (PMC8420972; doi:10.1155/2021/6203759)
Supplement: Supplementary 2 — Figure S1. The correlation between the promoter DNA methylation and gene expression levels of TNFRSF4, LCORL, and PLAU in the validation datasets (A: GSE41117 and B: GSE75539). [file 6203759.f2.pdf]

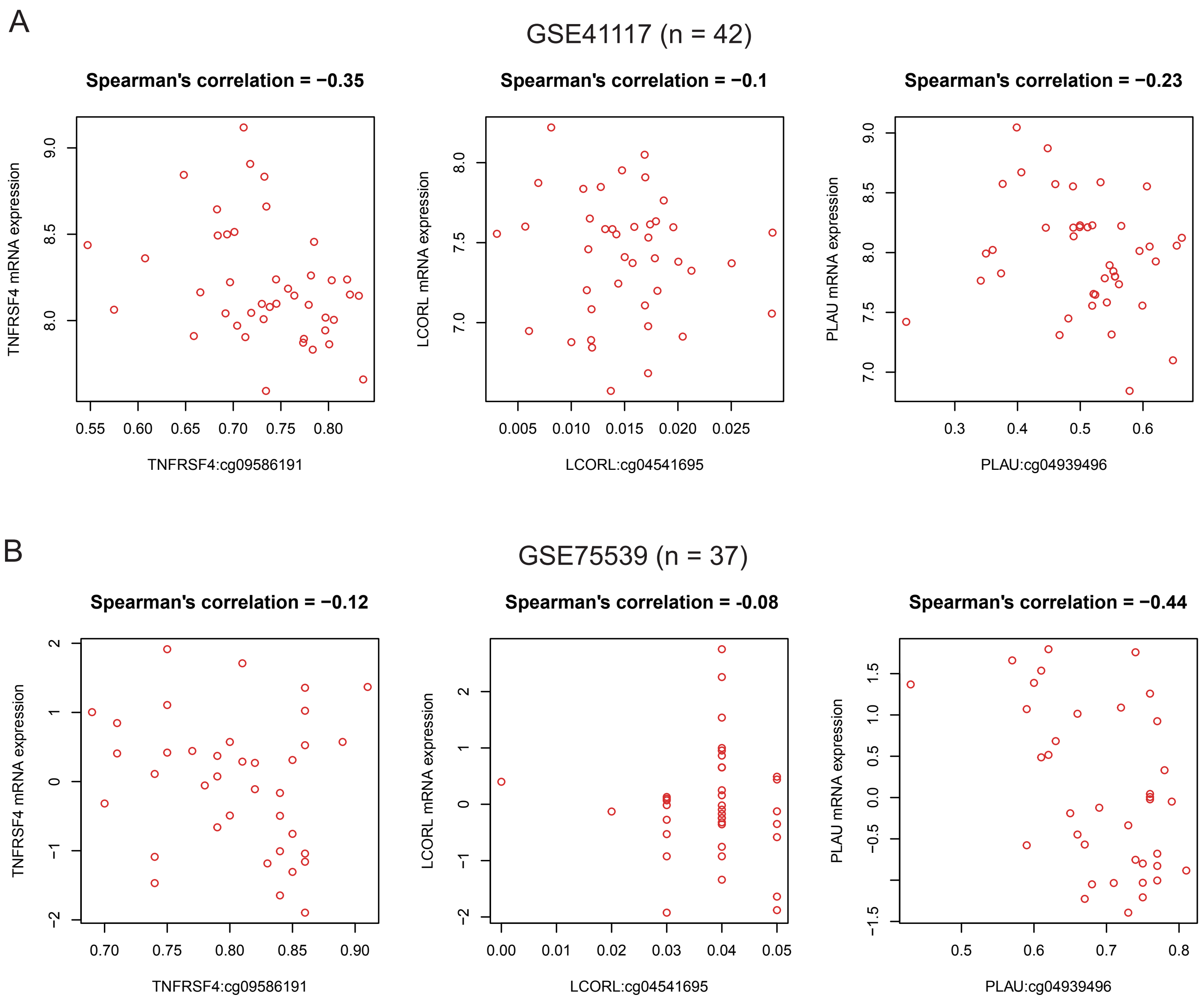
A
GSE41117 (n = 42)
Spearman's correlation = −0.35
TNFRSF4 mRNA expression
TNFRSF4:cg09586191
Spearman's correlation = −0.1
LCORL mRNA expression
LCORL:cg04541695
Spearman's correlation = −0.23
PLAU mRNA expression
PLAU:cg04939496
B
GSE75539 (n = 37)
Spearman's correlation = −0.12
TNFRSF4 mRNA expression
TNFRSF4:cg09586191
Spearman's correlation = -0.08
LCORL mRNA expression
LCORL:cg04541695
Spearman's correlation = −0.44
PLAU mRNA expression
PLAU:cg04939496
